# Molecular and Cellular Bases of Lipodystrophy Syndromes

**DOI:** 10.3389/fendo.2021.803189

**Published:** 2022-01-03

**Authors:** Jamila Zammouri, Camille Vatier, Emilie Capel, Martine Auclair, Caroline Storey-London, Elise Bismuth, Héléna Mosbah, Bruno Donadille, Sonja Janmaat, Bruno Fève, Isabelle Jéru, Corinne Vigouroux

**Affiliations:** ^1^ Sorbonne University, Inserm UMR_S 938, Saint–Antoine Research Centre, Cardiometabolism and Nutrition University Hospital Institute (ICAN), Paris, France; ^2^ Endocrinology Department, Assistance Publique–Hôpitaux de Paris, Saint–Antoine Hospital, National Reference Centre for Rare Diseases of Insulin Secretion and Insulin Sensitivity (PRISIS), Paris, France; ^3^ Assistance Publique–Hôpitaux de Paris, Robert Debré Hospital, Pediatric Endocrinology Department, National Competence Centre for Rare Diseases of Insulin Secretion and Insulin Sensitivity (PRISIS), Paris, France; ^4^ Genetics Department, Assistance Publique–Hôpitaux de Paris, La Pitié-Salpêtrière Hospital, Paris, France

**Keywords:** lipodystrophy, insulin resistance, diabetes, adipose tissue, genetics, senescence, lipomatosis, immunity

## Abstract

Lipodystrophy syndromes are rare diseases originating from a generalized or partial loss of adipose tissue. Adipose tissue dysfunction results from heterogeneous genetic or acquired causes, but leads to similar metabolic complications with insulin resistance, diabetes, hypertriglyceridemia, nonalcoholic fatty liver disease, dysfunctions of the gonadotropic axis and endocrine defects of adipose tissue with leptin and adiponectin deficiency. Diagnosis, based on clinical and metabolic investigations, and on genetic analyses, is of major importance to adapt medical care and genetic counseling. Molecular and cellular bases of these syndromes involve, among others, altered adipocyte differentiation, structure and/or regulation of the adipocyte lipid droplet, and/or premature cellular senescence. Lipodystrophy syndromes frequently present as systemic diseases with multi-tissue involvement. After an update on the main molecular bases and clinical forms of lipodystrophy, we will focus on topics that have recently emerged in the field. We will discuss the links between lipodystrophy and premature ageing and/or immuno-inflammatory aggressions of adipose tissue, as well as the relationships between lipomatosis and lipodystrophy. Finally, the indications of substitutive therapy with metreleptin, an analog of leptin, which is approved in Europe and USA, will be discussed.

## Introduction

Lipodystrophy syndromes are rare diseases characterized by generalized or segmental lack of adipose tissue, and by insulin resistance-related metabolic complications such as diabetes, hypertriglyceridemia, hepatic steatosis, and ovarian hyperandrogenism in women. Besides their different clinical presentation with generalized or partial lipoatrophy, accompanied or not by fat overgrowth in other body areas, lipodystrophy syndromes are highly heterogeneous diseases in several other aspects. Specific subtypes of lipodystrophy are associated with additional clinical signs and complications, with, among others, neurological or cardiovascular involvement, showing that lipodystrophy syndromes are frequently complex multisystem diseases (1–3). The onset of lipodystrophy may be precocious, in early infancy, or delayed in late childhood or adulthood. This review will mainly focus on genetic forms of lipodystrophies. Other forms of lipodystrophies, that will not be covered by this review, result from iatrogenic therapies and/or other environmental factors. This is the case for HIV-related lipodystrophies, due to multifactorial mechanisms resulting, among others, from HIV infection and antiretroviral agents (4). Glucocorticoid therapy leads to body fat redistribution and insulin resistance (5). The identification of causative pathogenic variants in more than 20 genes leading to monogenic forms of lipodystrophies has highlighted several determinants of adipose tissue pathophysiology. This field of research, still highly productive, indicates adipose tissue as a major actor to ensure proper whole-body insulin sensitivity (3, 6).

## Main Molecular Causes of Lipodystrophy Syndromes and Their Impact on Adipose Tissue Functions and Insulin Response

### Main Molecular Causes of Lipodystrophy Syndromes

**Figure 1:**
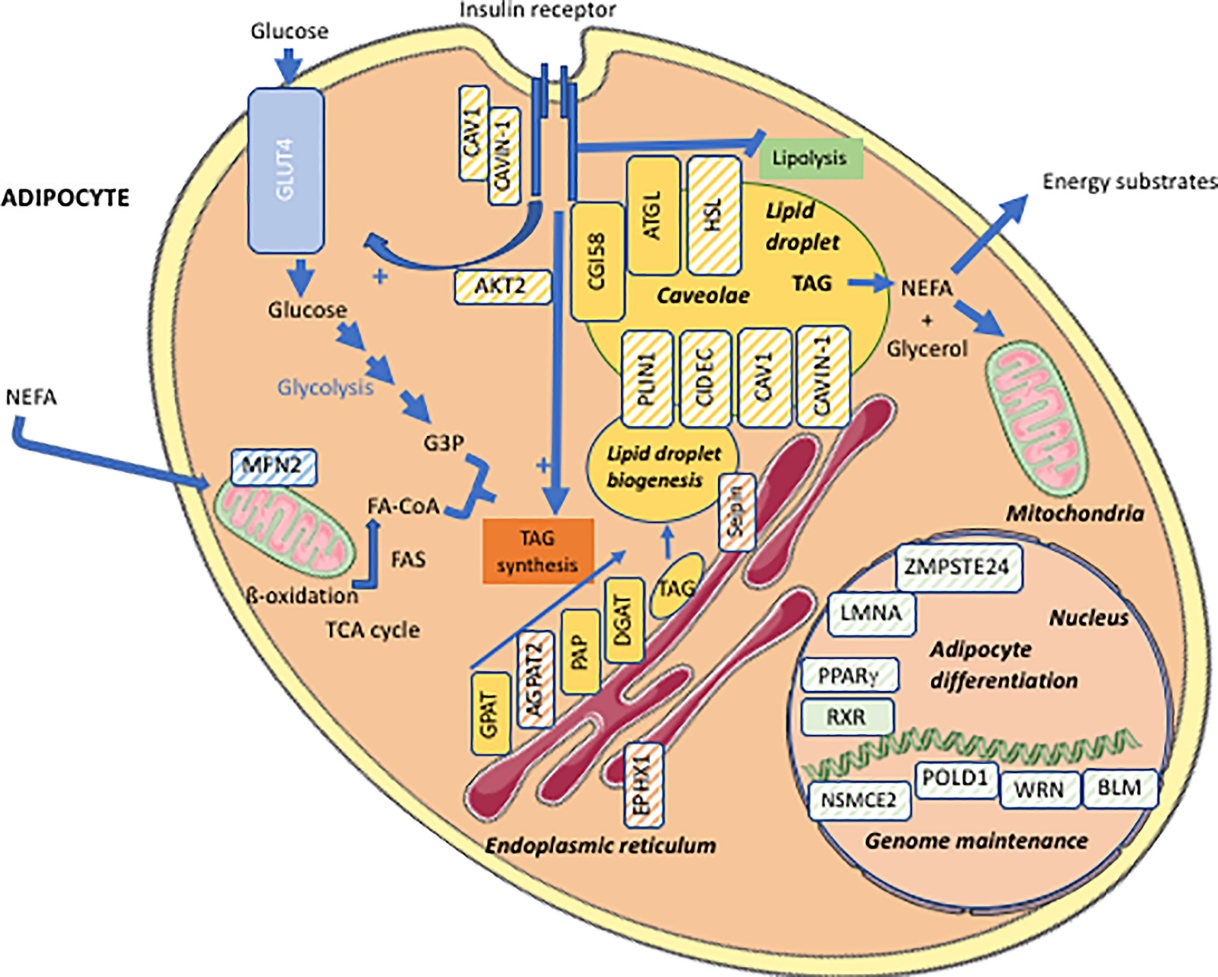
Cellular targets of the main molecular defects responsible for lipodystrophy syndromes. Adipocyte schematic representation with localization of the main proteins involved in the molecular pathophysiology of lipodystrophy syndromes (hatched symbols). AGPAT2, 1-acylglycerol-3-phosphate-O-acyltransferase 2; AKT2, serine/threonine-protein kinase 2; ATGL, adipose triglyceride lipase; BLM, Bloom syndrome protein; CAV1, caveolin-1; CAVIN1, cavin-1; CGI58, comparative gene identification-58, also known as α/β-hydrolase domain-containing 5 (ABHD5); DGAT, diacylglycerol acyltransferase; EPHX1, epoxide hydrolase 1; FA, fatty acid; FA-CoA, fatty acid-coenzyme A; FAS, fatty acid synthase; G3P, glycerol-3-phosphate; GLUT4, glucose transporter 4; GPAT, glycerol-3-phosphate acyltransferase; HSL, hormone-sensitive lipase; LMNA, lamin A/C; MFN2, mitofusin-2; NEFA, non-esterified fatty acids; NSMCE2, E3 SUMO-protein ligase NSE2; PAP, phosphatidic acid phosphatase; PLIN1, perilipin-1; POLD1, DNA polymerase delta 1, catalytic subunit; PPARγ, peroxisome proliferator-activated receptor gamma; RXR, retinoid X receptor; TAG, triacylglycerol; TCA cycle, tricarboxylic acid cycle; WRN, WRN RecQ like helicase.

Lipodystrophy syndromes include different congenital to adult-onset diseases, with either generalized or partial lipoatrophy. More than 20 genes are involved in monogenic lipodystrophy syndromes (6–8). Although lipodystrophy syndromes have been considered as ultra-rare diseases, with a prevalence of less than 5 cases per million (9), they are largely underdiagnosed, and systematic genetic screening suggests that 1/7000 individuals could be affected, with a majority of partial forms (10). **Table 1** indicates the main monogenic lipodystrophy syndromes, their specific phenotypic features and the main functions of involved genes. The diversity of molecular causes of lipodystrophy reflects both clinical heterogeneity and close pathophysiological relationships of these diseases. Indeed, beyond the diversity of clinical forms, lipodystrophy syndromes share adipose tissue dysfunction as a key pathophysiological feature, with gene pathogenic variants mostly affecting adipocyte development, differentiation and/or functions (**Figure 1**).

**Table 1:** Main forms of monogenic lipodystrophy syndromes.

TYPE OF LIPODYSTROPHY	TRANSMISSION	SPECIFIC FEATURES ASSOCIATED WITH LIPODYSTROPHY	GENE INVOLVED, MAIN CELLULAR FUNCTIONS
**Generalized lipodystrophy syndromes**			
**CGL1**	AR	Lytic bone lesions, cardiomyopathy	*AGPAT2*: AGPAT2, synthesis of adipocyte triglycerides and glycerophospholipids
**CGL2**	AR	Intellectual deficiency, cardiomyopathy, rare neurological signs (encephalopathy, spasticity)	*BSCL2*: seipin, formation of adipocyte lipid droplet
**CGL3**	AR	Short stature, megaesophagus	*CAV1*: caveolin-1, intracellular transduction pathways, lipid droplet wall
**CGL4**	AR	Muscular dystrophy, cardiac conduction abnormalities, achalasia	*CAVIN1*: cavin-1, partner of caveolin-1
**Autoinflammatory lipodystrophy** **(JMP, CANDLE)**	AR	Fever, muscle atrophy, systemic skin and joint inflammation	*PSMB8*: Immunoproteasome subunit PSMB8, regulation of interferon production, and protein quality control Genes encoding other immuno-proteasome subunits have also been involved
**Partial lipodystrophy syndromes**			
**FPLD2**	AD	Cushingoid facies, possible association with skeletal and cardiac muscular dystrophy	*LMNA:* lamin A/C, structure and functions of nucleus
**FPLD3**	AD	Severe hypertension	*PPARG:* PPARγ, adipocyte differentiation
**FPLD4**	AD	Acromegaloid features	*PLIN*: perilipin-1, structure and function of lipid droplet
**FPLD5**	AR	–	*CIDEC:* CIDEC, structure and function of lipid droplet
**AKT2- linked lipodystrophy**	AD	Insulin-resistant diabetes with moderate lipodystrophy	*AKT2:* Akt2, insulin signaling intermediate
**Partial lipodystrophy syndromes with pseudo-lipomatosis/Launois-Bensaude multiple lipomatosis**			
**FPLD6 (*LIPE*-linked lipodystrophy)**	AR	Upper-body fat overgrowth (pseudo-lipomatosis), lipoatrophy of limbs, insulin resistance-related traits, muscular atrophy in some cases	*LIPE:* Hormone-sensitive-lipase, release of fatty acids from stored triglycerides in adipocytes and release of cholesterol from cholesterol esters in steroidogenic tissues
***MFN2*-linked lipodystrophy**	AR	Pseudo-lipomatosis, lipoatrophy of non-lipomatous regions, axonal polyneuropathy	*MFN2:* Mitofusin-2, mitochondrial fusion
**Progeroid lipodystrophies**			
**Hutchinson-Gilford progeria syndrome**	*De novo*	Progeria: generalized lipoatrophy, growth retardation, dysmorphic signs, alopecia, bone and skin abnormalities, severe atherosclerosis in childhood	*LMNA:* lamin A/C, structure and functions of nucleus
**Progeroid laminopathies**	AD or *de novo*	Lipodystrophy with progeroid signs	*LMNA:* lamin A/C, structure and functions of nucleus
**Type A mandibuloacral dysplasia**	AR	Partial lipodystrophy with progeroid signs and mandibular involvement	*LMNA:* lamin A/C, structure and functions of nucleus
**Type B mandibuloacral dysplasia**	AR	Generalized lipodystrophy with progeroid signs and mandibular involvement	*ZMPSTE24:* ZMPSTE24/FACE1, post-translational prelamin A maturation
**Neonatal progeroid syndrome**	AR or *de novo*	Generalized lipoatrophy, progeroid signs, other signs depending on the gene affected	*LMNA, ZMPSTE24:* maturation of prelamin A*CAV1:* caveolin-1, intracellular transduction pathways, structure of lipid droplet
**Werner syndrome (adult onset progeria)**	AR	Cataracts, trophic skin disorders, cancers, subcutaneous lipoatrophy and increased perivisceral fat	*WRN:* WRN, DNA helicase, DNA repair
**MDPL (Mandibular hypoplasia, Deafness and Progeroid features syndrome with Lipodystrophy)**	*De novo*	Subcutaneous lipoatrophy and increased perivisceral fat, acro-osteolysis, mandibular and clavicular dysplasia, deafness	*POLD1:* DNA polymerase delta 1, catalytic subunit, DNA replication and repair
***NSMCE2*-linked lipodystrophy**	AR	Short stature, hypogonadism, extreme insulin resistance	*NSMCE2*: E3 SUMO-protein ligase NSE2, genome maintenance, DNA repair
***EPHX1*-linked lipoatrophy**	AR	Generalized lipoatrophy, dysmorphic and progeroid signs, hepatic cytolysis, sensorineural hearing loss	*EPHX1:* Epoxyde hydrolase 1, hydrolysis of epoxides to less-reactive diols

Type 1 Familial Partial Lipodystrophy (FPLD1) is a multigenic form of lipodystrophy syndrome with exacerbated android morphotype and predominant limb lipoatrophy.

AD, autosomal dominant; AGPAT2, 1-Acylglycerol-3-Phosphate-O-Acyltransferase 2; AR, autosomal recessive; CGL, congenital generalized lipodystrophy; JMP, Joint contractures, Muscular atrophy, Microcytic anemia and Panniculitis-induced lipodystrophy syndrome; CANDLE, Chronic atypical neutrophilic dermatosis with lipodystrophy and elevated temperature syndrome; FPLD, familial partial lipodystrophy.

**Figure 2:**
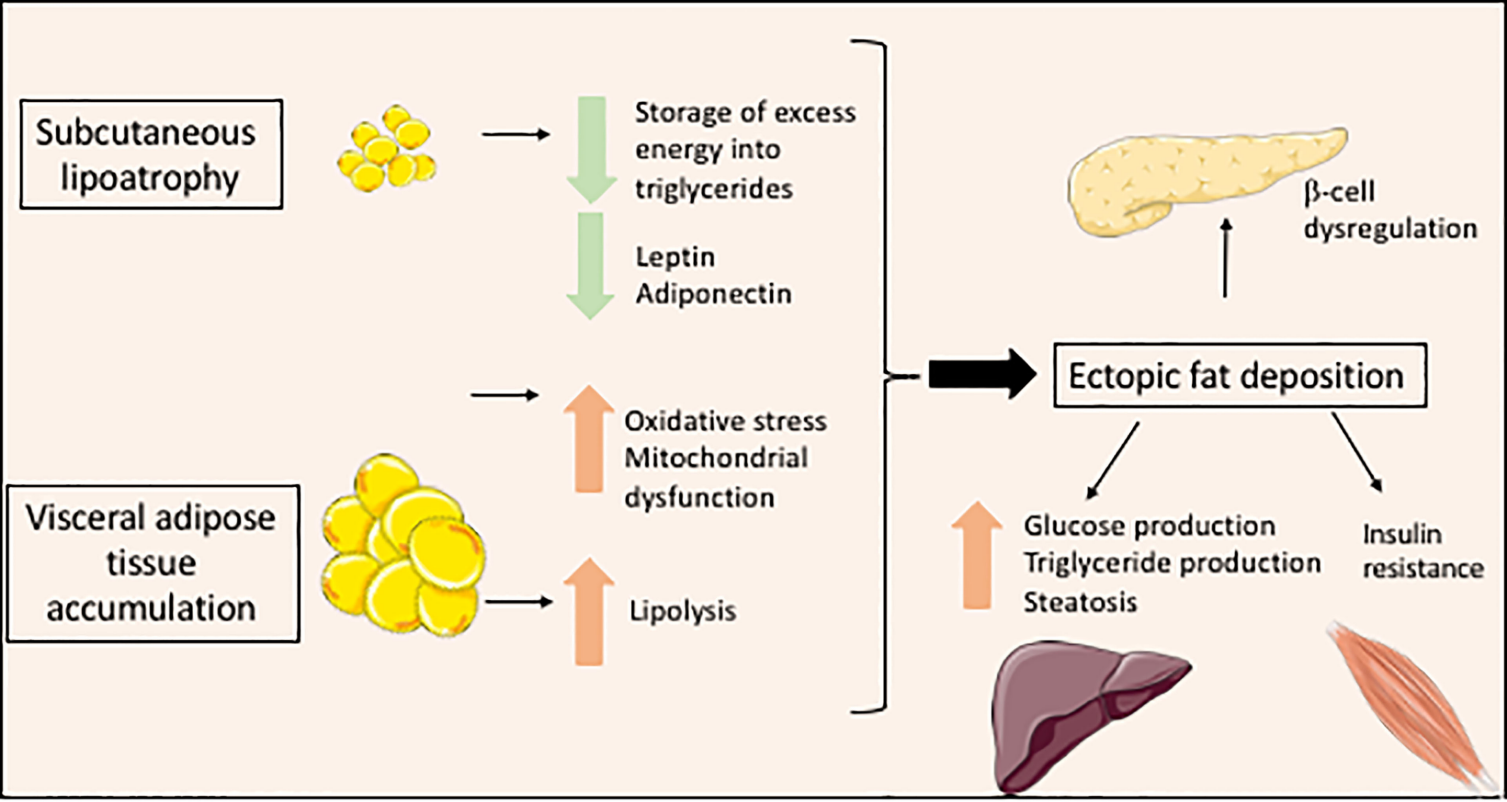
Metabolic consequences of lipodystrophy leading to cellular lipotoxicity.

Congenital generalized lipodystrophy syndromes (CGL or Berardinelli-Seip Congenital Lipodystrophy) are autosomal recessive diseases, mainly observed in patients from consanguineous families. They are mainly due to null variants in *AGPAT2* encoding 1-acylglycerol-3-phosphate-O-acyltransferase 2, involved in triglyceride and phospholipid synthesis, or in *BSCL2* encoding seipin, an endoplasmic reticulum membrane protein which contributes to lipid droplet biogenesis (11–16). CGL3 and CGL4 are due to genetic defects in caveolin-1 or cavin-1 respectively, involved in the formation of cell plasma membrane microdomains called caveolae, that initiate several signaling pathways. Caveolin-1 and cavin-1 are also localized at the adipocyte lipid droplet and contribute to intracellular fluxes of lipids (17, 18) (**Figure 2**). Most familial partial lipodystrophies (FPLD) are transmitted as autosomal dominant diseases due to loss-of-function or dominant negative mutations, with initial clinical manifestations occurring from late childhood onwards. Apart FPLD1, which is probably a multigenic form of lipodystrophy syndrome (19), FPLD2 due to *LMNA* pathogenic variants, is the most frequent form of genetically determined partial lipodystrophy (10). Generalized or partial lipoatrophy is a clinical feature of several diseases with accelerated ageing called progeroid syndromes (**Table 1**).

### Lipodystrophy and Lipotoxicity

Lipoatrophy is a main contributor of metabolic complications associated with lipodystrophy syndromes. Adipocytes represent 20 to 40% of the cell population of adipose tissue and 90% of adipose tissue volume (20, 21). They arise from mesenchymal stem cells, adipocyte differentiation being carried out under the control of several adipogenic transcription factors. PPARγ is a major adipogenic factor, which regulates the expression of several genes of lipid metabolism and modulates both insulin sensitivity and secretory functions of adipocytes (21, 22). White adipocytes are the most abundant adipocytes in humans. They are characterized by a single voluminous lipid vacuole surrounding a neutral lipid core mainly composed of triglycerides and cholesterol esters. The adipocyte lipid droplet is coated with a monolayer of phospholipids and with proteins belonging to the perilipin family, which play important structural and functional roles. Indeed, white adipocytes have a crucial role in the regulation of energy balance and systemic metabolic homeostasis. In response to nutritional and hormonal signals, the adipocyte lipid droplet is able to store excess energy as triglycerides in the postprandial state, and then to release fatty acids from triglycerides, providing energy substrates for other organs. Adequate storage of energy in adipocytes protects other organs from lipotoxicity due to lipid overload, which leads to oxidative stress, mitochondrial dysfunction, apoptosis and tissue dysfunction (23). In lipodystrophy syndromes, the very limited adipose tissue expandability, due to the major decrease in the capacity of adipose tissue to store lipids, exposes non-adipose organs to lipotoxicity even in well-balanced diet conditions. This results in muscle insulin resistance due to disruption of insulin signaling (24), in non-alcoholic fatty liver disease, as well as in myocardial, endothelial and pancreatic beta-cell dysfunction (25–27). Adipocytes are also important autocrine, paracrine, and endocrine cells that produce numerous adipokines. Among others, leptin regulates satiety by acting on hypothalamic neurons, and modulates carbohydrate and lipid metabolism by acting on muscle, liver adipose tissue and pancreatic beta-cells (28, 29). Integrated effects of leptin ensure an efficient protection of non-adipose tissues from lipid accumulation (30). Adiponectin produced by adipose tissue increases fatty acid oxidation and glucose transport in muscle, and decreases hepatic gluconeogenesis. In lipodystrophy syndromes, the lack of functional subcutaneous fat drives multiple metabolic alterations resulting from altered metabolic and secretory functions of adipocytes. Decreased adipose tissue expandability, and leptin deficiency induce ectopic accumulation of fat upon increased energy intake, even during the physiological postprandial state (**Figure 2**). Furthermore, decreased serum levels of adiponectin, although not to the same extent in the different forms of lipodystrophy (31), contribute to insulin resistance and hepatic steatosis associated with lipodystrophy (32). Altered production of other adipokines by lipodystrophic adipose tissue has also been described in several studies, mostly related to HIV-related forms of lipodystrophy (4). It could lead to adipose tissue inflammation and fibrosis, and to a state of systemic low-grade inflammation with insulin resistance (33). In lipodystrophy syndromes, insulin signaling pathways are strongly impacted by mechanisms linked to cellular lipotoxicity and metabolic inflexibility (23, 24). Increased lipid fluxes activate hepatic production of very-low-density lipoproteins, triglycerides and glucose, and impair muscular glucose uptake (6, 34, 35). Leptin deficiency also increases appetite, which worsens metabolic profile. In some forms of partial lipodystrophies, redistribution of body fat, with increased visceral fat and decreased subcutaneous fat, particularly of the lower limbs, also contributes to metabolic dysfunction. Indeed, subcutaneous adipose tissue is physiologically more sensitive to insulin. It has been shown that subcutaneous adipose tissue of the lower part of the body is protective against diabetes and cardiovascular diseases in the general population (36, 37). Conversely, visceral adipose tissue is more sensitive than subcutaneous adipose tissue to adipocyte lipolysis. Furthermore, visceral adipose tissue directly releases fatty acids into the portal vein during lipolysis, which are captured by the liver, leading to an increased risk of liver lipotoxicity, liver steatosis and insulin resistance (**Figure 2**). Visceral fat is more susceptible to chronic inflammation and fibrosis, and produces lower amounts of leptin than subcutaneous tissue (38). Excess visceral, but not subcutaneous fat, is involved in adiposity-related hypertension (39). Mitochondrial dysfunction and oxidative stress, which are frequently observed cellular consequences of lipodystrophy, also decrease cellular responses to insulin (40–42) (**Figure 2**).

## Main Clinical Features of Generalized and Partial Lipodystrophies

The diagnosis of lipodystrophy syndromes is based first and foremost on clinical examination. Since objective clinical measures are still lacking to document an abnormal development of subcutaneous fat, the clinical diagnosis of lipodystrophy is highly dependent on the clinician’s self-experience. It can be particularly difficult in men affected with partial lipodystrophy syndromes, in whom the diagnosis could be missed or substantially delayed. Indeed, the nosological framework is poorly defined between an android distribution of fat, commonly observed in the general population, which is a major risk factor for insulin resistance-related diseases (43), and the lipodystrophic phenotype. This is particularly striking for Type 1 Familial Partial Lipodystrophy (FPLD1), characterized by a central repartition of fat with lipoatrophy of limbs and severe insulin resistance, which is probably of polygenic origin (19). However, several clinical features are common to lipodystrophy syndromes, so that a distinctive clinical picture may be identified.

### Lipoatrophy

The identification of generalized or segmental lipoatrophy, in the absence of malnutrition, is a major step for diagnosis.

**Figure 3:**
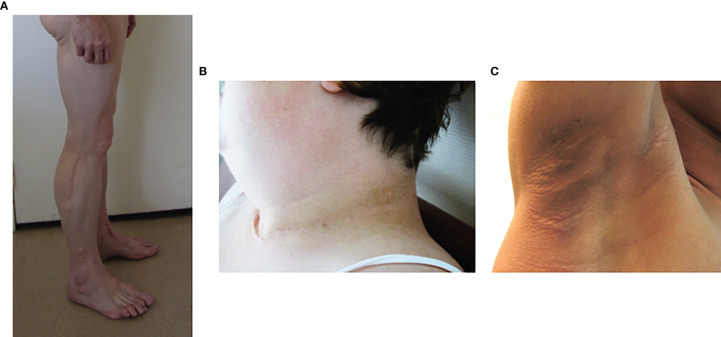
Phenotypic features of lipodystrophy syndromes. **(A)** Muscular hypertrophy and lipoatrophy of limbs in Type 2 Familial Partial Lipodystrophy (Dunnigan syndrome). **(B, C)** Cervical and axillary *acanthosis nigricans* in patients with lipodystrophy due to *LMNA* **(B)** or *BSCL2* **(C)** pathogenic variants.

In generalized forms of lipodystrophy syndromes, lipoatrophy of the face is striking. The patient’ facies is emaciated due to the absence of Bichat fat pads. Acromegaloid features, with protruding eyebrow arches, cheekbones, and lower jaw, and thick facial traits, are also observed, especially in congenital forms of lipoatrophy, but also in some partial forms of the disease (8, 44). These clinical signs are due to an increased visibility of bone structures in the absence of adipose tissue, and to the stimulation of growth factor pathways by excess insulin. Hands and feet may also be thickened. The lack of subcutaneous adipose tissue also increases the visibility of muscles (athletic appearance) and of veins (pseudo-veinomegaly) in limbs, trunk and abdomen. In addition, it should be noted that, at least in some cases, the volume and mass of skeletal muscle are truly increased (45, 46) (**Figure 3A**). Ingrown toenails are possible consequences of severe lipoatrophy of feet. Hypomastia is common in women, secondary to loss of breast adipose tissue and/or to hyperandrogenism.

### Fat Accumulation

In most forms of partial lipodystrophy syndromes, lipoatrophy mainly affects lower limbs and can coexist with areas of fat accumulation. Dunnigan syndrome (FPLD2), due to pathogenic variants in the *LMNA* gene encoding Type A lamins, is characterized by lipoatrophy of limbs and trunk with cushingoid features of face and neck, *i.e.* increased supraclavicular fat pads, double chin, buffalo hump, and with perineal accumulation of adipose tissue. The general morphotype is strikingly android, with a biacromial diameter greater than the bitrochanteric diameter (8, 47). Muscle hypertrophy, contrasting with the usual amyotrophy associated with hypercortisolism, may suggest the diagnosis of lipodystrophy syndrome (**Figure 3A**). Fat overgrowth may lead to massive pseudo-lipomatous regions in upper body and proximal limb areas, contrasting with lipoatrophy of non-lipomatous regions, in specific genetic forms of partial lipodystrophy (48–55). On the contrary, in Barraquer-Simons acquired partial lipodystrophy syndrome, a progressive lipoatrophy develops in upper parts of the body (face, trunk and upper limbs), while adipose tissue accumulates in lower limbs.

### Insulin Resistance-Associated Clinical Signs

*Acanthosis nigricans*, a brownish hyperkeratotic thickening of the skin, and acrochorda (skin tags), are very common clinical signs of insulin resistance in lipodystrophy syndromes (**Figures 3B, C**). These skin lesions are usually located in cervical, axillary and inguinal folds, but may be very extensive in some patients. Insulin resistance frequently leads to ovarian hyperandrogenism in women, with hirsutism, irregular menses and polycystic ovaries (47, 56, 57). Hepatomegaly, resulting from dysmetabolic liver steatosis, is common. Hypertriglyceridemia can be complicated, or even revealed, by acute pancreatitis (7, 58).

### Cardiovascular Signs

High blood pressure is frequent, and can be very severe, in particular in partial lipodystrophies associated with pathogenic variants of *PPARG* encoding the adipogenic factor PPARγ (peroxisome proliferator-activated receptor gamma) (59–61). The risk of atherosclerosis is strongly increased, which could result from insulin resistance, diabetes, and hypertension (62), but also from genetic variants that directly target the vascular wall, as observed in *LMNA*-related lipodystrophies (40, 63, 64). In addition to non-specific diabetic cardiomyopathy and atherosclerosis, several cardiovascular complications can be observed in the different forms of lipodystrophies. Patients with congenital generalized lipoatrophy may suffer from hypertrophic cardiomyopathy, with or without hypertension, associated with ectopic cardiac fat and/or lipotoxicity (65, 66). Pathogenic variants in *LMNA* are responsible for lipodystrophy syndromes with early atherosclerosis and/or with dilated cardiomyopathy, rhythm and/or conduction disorders and/or extensive calcifications of cardiac valves (67–72). A regular cardiovascular screening, with cardiac ultrasound and stress test, and, if needed, coronary CT angiogram, 24-hour ECG monitoring, and/or cardiac MRI is required in most patients with lipodystrophy syndromes (7, 8).

### Other Clinical Signs

Depending on their molecular cause, lipodystrophy syndrome can be associated with several other clinical signs. Moderate intellectual disability can be observed in type 2 Congenital Generalized Lipoatrophy (CGL) due to *BSCL2* pathogenic variants encoding seipin (11). Digestive signs are frequent in neonates or infants with CGL. In late infancy or adolescence, dysphagia can reveal megaesophagus, due to esophageal achalasia, in CGL due to pathogenic variants of *CAVIN1* or *CAV1*, encoding proteins involved in the formation of caveolae at the cell plasma membrane (73–77). Growth disorders, dysmorphic features with micrognathia, beaked nose, dental crowding, prominent eyes, dystrophic bones, and/or signs suggesting accelerated aging such as precocious whitening and/or loss of hair, sclerodermatous skin appearance, joint limitations, and/or muscle atrophy are hallmarks of progeroid lipodystrophies (67, 78, 79). Other signs such as precocious cataracts, trophic skin disorders, hypogonadism, predisposition to cancer can be associated in progeroid lipodystrophy syndromes due to defects in DNA repair (80, 81). Muscle functional defects are observed in some patients with lipodystrophy due to pathogenic variants in *LMNA*, *CAVIN1*, or *PSMB8*, among other genes (67, 74, 82–85). Systemic inflammatory signs (fever, multiorgan inflammatory involvement affecting joints, skin, heart) are prominent features of lipodystrophies associated with rare autoinflammatory syndromes of genetic origin (86). The occurrence of some lipodystrophy syndromes in the context of panniculitis or autoimmune diseases, suggests that adipose tissue could be a target of immune tolerance disruption (87).

## Biological Characteristics of Lipodystrophy Syndromes

Insulin resistance, resulting from adipose tissue dysfunction and subsequent ectopic lipid deposition, is one of the hallmarks of lipodystrophy syndromes. High serum insulin levels, with normal or increased plasma glucose, are detected in the fasting state and/or during oral glucose tolerance test. Patients with diabetes display preserved or even increased C-peptide levels for a long time, and/or frequently require very high doses of insulin to achieve glucose control. Hypertriglyceridemia is also very frequent, and can reach very high values, associated with a high risk of acute pancreatitis. Low HDL-cholesterol is almost always present. Increased liver enzymes are also common features, due to liver steatosis or fibrosis. Biological signs of adipose dysfunction include decreased serum adiponectin, and either barely detectable leptin levels in generalized lipodystrophies, or lower leptin levels than predicted by body mass index in partial lipodystrophies. Creatine phosphokinase may be elevated, especially in patients with lipodystrophies and muscular dystrophy. Ovarian hyperandrogenism results from insulin resistance, with high levels of total and free testosterone and of Δ4-androstenedione, decreased sex-hormone binding globulin, and increased luteinizing hormone to follicle-stimuling hormone ratio (7, 8).

## Imaging Investigations

**Figure 4:**
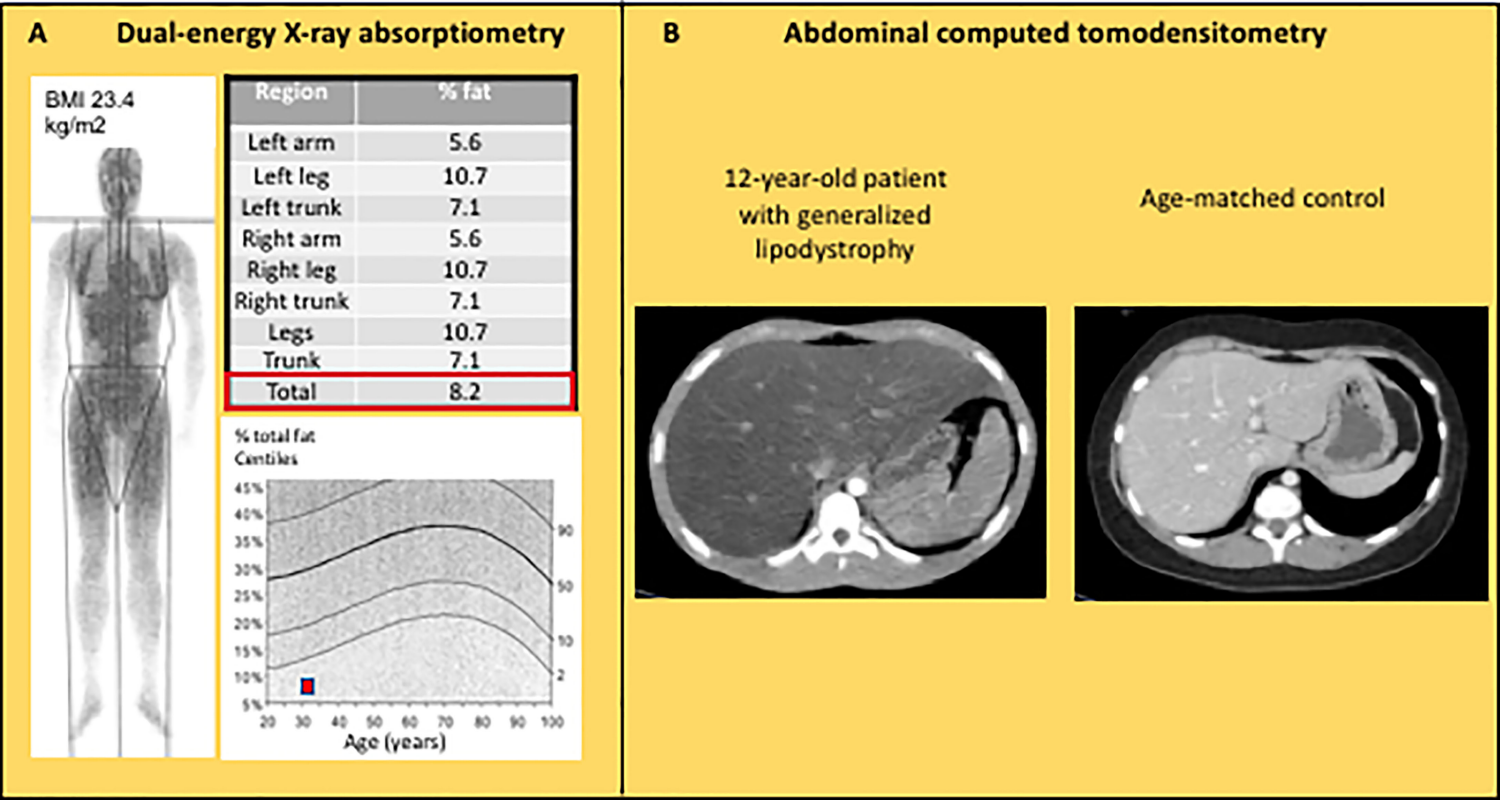
Imaging features in lipodystrophy syndromes. **(A)** Dual energy-ray absorptiometry (DEXA) in a 31 year-old patient with CGL1, showing a major decrease in total and segmental fat mass. **(B)** Abdominal computed tomodensitometry in a 12 year-old patient with acquired generalized lipodystrophy showing homogeneous hepatomegaly with low attenuation of the parenchyma (Hounsfield units: -13), and absence of subcutaneous adipose tissue.

Although the diagnosis of lipodystrophy is mainly based on clinical examination, the objective measurement of fat mass by dual energy-ray absorptiometry (DEXA) is useful to determine the severity of lipoatrophy and, in partial forms of lipodystrophies, to document the altered segmental distribution of fat (**Figure 4A**). Abdominal ultrasound, computed tomodensitometry (CT), or magnetic resonance imagery (MRI) are required to search for liver steatosis and signs of cirrhosis (**Figure 4B**). In women, pelvic ultrasound may reveal ovaries of increased volume and/or with multiple follicles. Bone imaging can show precocious non-specific degenerative radiographic abnormalities in patients with familial partial lipodystrophies. Several bone lesions such as osteolysis, osteosclerosis or pseudo-osteopoikilosis are frequently present in generalized forms of lipodystrophy, and may lead to misdiagnosis (88, 89). Muscle imaging (CT or MRI) may show muscular hypertrophy and/or fatty degeneration, with lack of subcutaneous adipose tissue. Imaging investigations may be completed by an electromyogram to search for neuropathic and/or myopathic signs.

## Lipodystrophy and Ageing

Remodeling of body fat occurs during physiological ageing, with decrease in subcutaneous gluteofemoral adipose tissue, increase in visceral fat depots and ectopic deposition of lipids. Together with pro-inflammatory changes in adipose tissue, these lipodystrophy-like features contribute to age-related insulin resistance (90).

Accelerated ageing is probably one of the most important pathophysiological mechanisms of primary lipodystrophies. Arguments in favor of this hypothesis first came from studies of *LMNA*-associated diseases. *LMNA* encodes Type A lamins, nuclear proteins that interact with chromatin and regulate several nuclear functions including epigenetic cell developmental programs (91) (**Figure 1**). *LMNA* pathogenic variants cause several different laminopathies including muscular dystrophy, cardiomyopathy, neuropathy, lipodystrophy, and syndromes of accelerated aging (progeria and progeroid syndromes). Both typical FPLD2/Dunnigan syndrome, characterized by partial lipodystrophy and insulin resistance-related complications, and the extremely severe Hutchinson-Gilford accelerated ageing syndrome with generalized lipoatrophy are due to *LMNA* variants. In addition to lipodystrophy, several other clinical features, although of very different severity, are part of the clinical spectrum of both diseases. This is the case for early atherosclerosis, leading to frequent cardiovascular events before the age of 50 in patients with FPLD2, and to death at a mean age of 15 in Hutchinson-Gilford progeria (63, 92). In Hutchinson-Gilford progeria, but also in FPLD2, early atherosclerosis is due not only to metabolic risk factors, but also to direct pro-senescent effects of *LMNA* pathogenic variants on endothelial and vascular smooth muscle cells (40, 63, 93, 94). Clinical features of accelerated ageing are observed in patients with complex progeroid forms of *LMNA*-linked lipodystrophies, with a large continuum of severity between Dunnigan syndrome and Hutchinson-Gilford progeria (67, 71). At the cellular level, *LMNA* pathogenic variants impair the fate of several mesodermal lineages, such as endothelial vascular cells (64), myoblasts (95), cardiomyocytes (96), and adipocytes (97–101), and are involved in several signaling pathways which accelerate aging processes (102, 103).

The relationships between lipodystrophy and increased cellular senescence were further demonstrated by the identification of several pathogenic variants in genes involved in DNA repair as molecular causes of lipodystrophy syndromes (**Table 1** and **Figure 1**). As discussed above, lipodystrophies due to genetic alterations in DNA repair are clinically expressed as progeroid syndromes, with lipodystrophy, insulin resistance and related metabolic alterations, and signs of premature ageing. However, additional clinical features, specific to each different genetic subtype, may be part of the phenotype. Among others, cataracts, which are not part of the phenotype of *LMNA*-associated progeroid syndromes, are a typical feature of Werner progeria syndrome (81) and MDPL (Mandibular hypoplasia, Deafness, Progeroid features, and Lipodystrophy) syndrome (80, 104, 105). These diseases are due, respectively, to biallelic pathogenic variants in *WRN* encoding the WRN DNA helicase and exonuclease, and to heterozygous loss-of-function variants in *POLD1*, encoding a catalytic subunit of DNA polymerase δ, both enzymes playing a major role in maintaining genome stability. Lipodystrophy is associated with a predisposition to cancer in Werner syndrome, as well as in Bloom syndrome, due to mutations in *BLM* encoding a DNA helicase, or in ataxia-telangiectasia with altered DNA synthesis and excision-repair pathways (106). Cultured fibroblasts from patients with Werner or MDPL syndromes present signs of premature senescence (81, 107). Importantly, premature senescence was shown to impair adipogenesis in human pluripotent stem cells lacking either WRN or BLM helicases (108). Several other DNA replication/repair-associated lipodystrophies have been described (7, 78, 109, 110), frequently associated with short stature, hypogonadism, and trophic skin disorders, among other progeroid signs.

Premature senescence and oxidative stress, directly resulting from defects in genes involved in cellular ageing and/or genome stability, or from consequences of other genetic defects impacting adipocytes (79), are thus probably important pathophysiological determinants of lipodystrophies.

## Lipodystrophy and Immuno-Inflammatory Diseases

Lipodystrophy syndromes can develop during the course of systemic immune and/or inflammatory diseases, suggesting that adipose tissue dysfunction could result from auto-immune and/or inflammatory damages. This is the case in CANDLE (Chronic Atypical Neutrophilic Dermatosis with Lipodystrophy and Elevated temperature) syndrome and related auto-inflammatory genetic diseases, due to genetic defects in immunoproteasome subunits (86, 111). Dysregulation of the interferon pathway is a key pathophysiological link between the molecular causes of these diseases and their clinical expression (112). Lipodystrophy with metabolic complications is also part of the phenotype of several autoimmune diseases. Generalized lipodystrophy with severe hyperinsulinemia and leptin deficiency has been described in a child with APECED (Autoimmune PolyEndocrinopathy-Candidiasis-Ectodermal Dystrophy) due to pathogenic variant in *AIRE* resulting in the disruption of immune tolerance (113). The term “acquired lipodystrophy”, which designates lipodystrophy syndromes without any known genetic cause, underlies several auto-immune diseases with adipose tissue involvement (114–116). Autoantibodies directed against perilipin-1, an important lipid droplet protein which regulates lipolysis, could be involved in adipocyte dysfunction (87). Barraquer-Simons partial lipodystrophy syndrome, which predominantly affects women, is associated in one third of the cases with decreased C3 and/or C4 complement factors, and with membranoproliferative glomerulopathy due to C3 nephritic autoantibodies (117, 118).

Recently, lipodystrophy syndromes have been reported during the course of targeted cancer therapy using immune checkpoint inhibitors (119–122). These agents, by releasing nonspecific immunosuppressive pathways, are known to lead to multiorgan auto-immune related adverse events. Inflammatory features and infiltration of adipose tissue with CD3^+^ and/or cytotoxic CD8^+^ lymphocytes have been identified in anti-PD1-related lipodystrophy syndromes, strongly suggesting that disrupted immune tolerance to adipocyte self-antigens could be the cause of lipodystrophy (119–122).

To note, HIV-related lipodystrophies which are reviewed elsewhere (4), also result from altered adipocyte differentiation and inflammatory dysregulation (123).

## Lipodystrophy and Lipomatosis: Some Shared Pathophysiological Mechanisms?

In specific genetic forms of partial lipodystrophy, due to *MFN2* or *LIPE* biallelic pathogenic variants, fat overgrowth may lead to massive pseudo-lipomatous regions in upper body and proximal limb areas, leading to the diagnosis of multiple symmetric lipomatosis. However, recent studies have shown that fat accumulates in non-encapsulated pseudo-lipomas, which differ from the organization of typical lipomas, and that patients also present with lipoatrophy of non-pseudo-lipomatous region and with lipodystrophy-related metabolic complications. Therefore, multiple symmetric lipomatosis could be, at least in *MFN2* and *LIPE*-related forms, an exacerbated form of partial lipodystrophy (49–55).

*MFN2*-associated lipodystrophy is a mitochondrial disease due to biallelic pathogenic variants including a specific *MFN2* p.Arg707Trp substitution. *MFN2* encodes mitofusin-2, a transmembrane protein of the outer mitochondrial membrane whose oligomerization drives mitochondrial fusion (124) (**Figure 1**). Lipoatrophy, low serum leptin and adiponectin levels, as well as adipose tissue mitochondrial defects, oxidative stress and increased expression of some thermogenic markers, provide evidence of adipose tissue dysfunction in patients with *MFN2*-associated lipodystrophy (52, 54). Apart from lipodystrophy/pseudo-lipomatosis, patients may present with other clinical signs of mitochondrial involvement, including Charcot-Marie-Tooth peripheral neuropathy.

*LIPE* biallelic pathogenic variants may also lead to pseudo-lipomatous forms of partial lipodystrophy (49, 50, 53, 55). *LIPE* encodes the key lipolytic enzyme hormone-sensitive lipase (HSL) (**Figure 1**), and *LIPE* pathogenic variants leading to lipodystrophy act through loss-of-function mechanisms. Functional studies using adipose stem cells have shown that defective lipolysis and impaired adipocyte differentiation, but also mitochondrial dysfunction, could contribute to pathophysiological mechanisms in *LIPE*-related lipodystrophy syndrome (55). Importantly, although the metabolic phenotype and potential lipodystrophy signs were not systematically investigated, some patients carrying mtDNA mutations responsible for the myoclonic epilepsy and ragged red fibers (MERRF) syndrome were also reported with multiple symmetric lipomatosis (125). Mitochondrial defects have also been described in the most frequent form of multiple symmetric lipomatosis, associated with excessive alcohol consumption (126).

Therefore, mitochondrial alterations could lead to both pseudo-lipomatous and/or lipodystrophic diseases. Whether other mitochondriopathies induce lipodystrophic diseases in humans, as shown in mice, requires further investigations (127).

## Metreleptin Treatment of Metabolic Complications Associated With Lipodystrophy Syndromes

Lipodystrophy syndromes are multi-tissue diseases, which require a multidisciplinary management. Regarding metabolic alterations, dietary measures and physical activity are very important therapeutic tools. Indeed, avoiding a positive energy balance leading to ectopic lipid infiltration is a major objective to prevent and/or treat metabolic alterations in the context of adipose tissue failure (7). To date, no medication has proven to be effective to cure lipoatrophy. Metformin is frequently used as a first-line pharmacological drug to decrease insulin resistance. Statins are frequently prescribed to reduce the cardiovascular risk, and fenofibrate can be added in case of insufficient response on triglycerides. Medium chain fatty acid supplementation is used to reduce hypertriglyceridemia in children. Very limited data are available regarding the effects of antidiabetic drugs in patients with lipodystrophic diabetes. Very high doses of insulin therapy are frequently used, due to severe insulin resistance.

**Figure 5:**
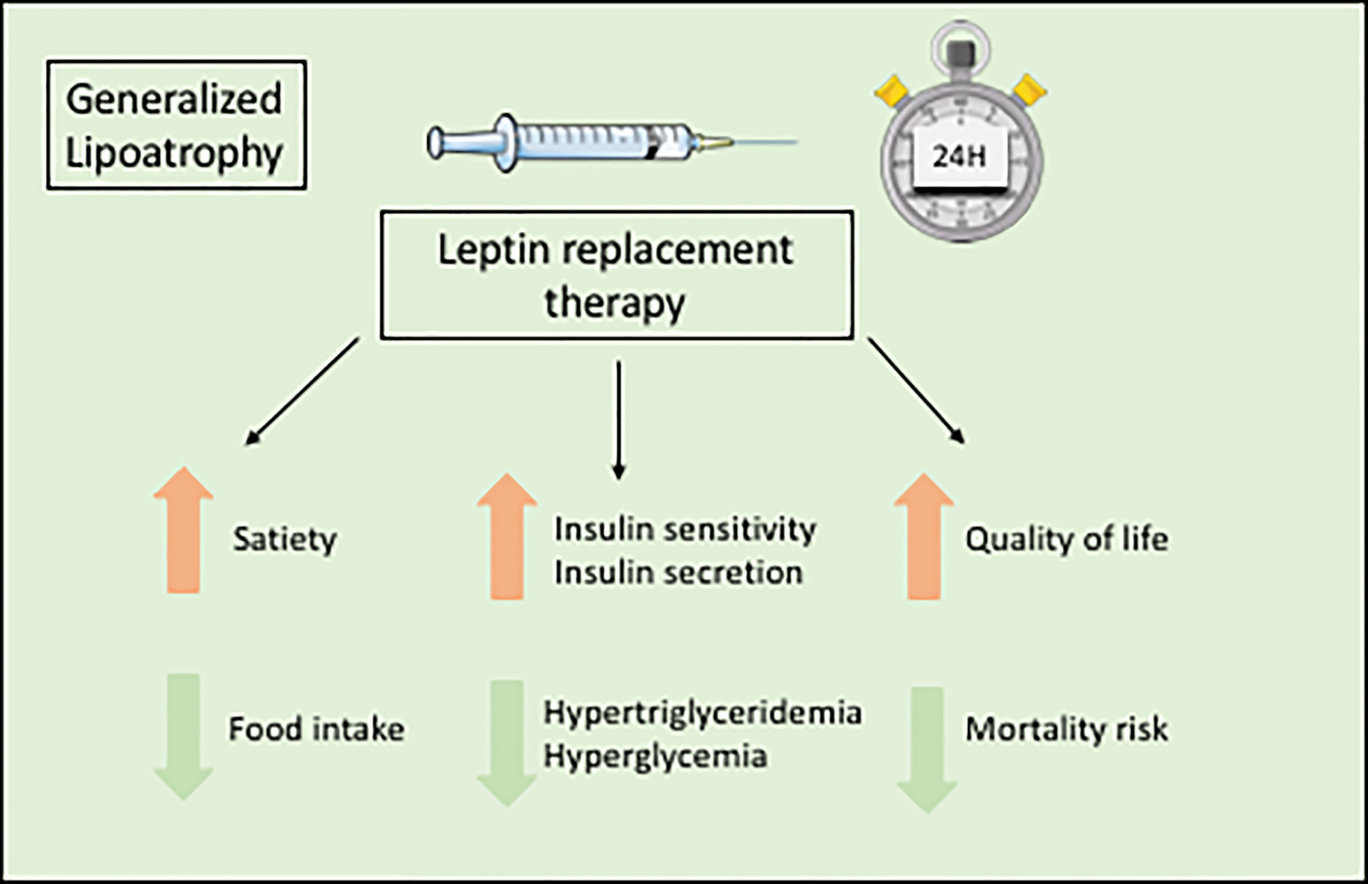
Benefits of metreleptin replacement therapy in generalized lipodystrophies.

The orphan drug metreleptin, a recombinant leptin analog, is the only specific treatment for the metabolic complications of lipodystrophy syndromes. Metreleptin, administered by subcutaneous injection once daily, is used as a hormone replacement therapy in patients with leptin deficiency. Metreleptin therapy obtained a marketing authorization to treat the complications of leptin deficiency in patients with lipodystrophy in Japan in 2013, in USA in 2014 (for generalized forms), and in Europe in 2018. Although metreleptin was not studied in placebo-controlled trials in the context of rare diseases, and although it does not lead to the reconstitution of lacking adipose tissue, it was shown effective, as an adjunct to diet, on metabolic and hepatic parameters in generalized lipodystrophy syndromes (**Figure 5**). In patients with lipodystrophy, metreleptin replacement therapy prevents ectopic storage of lipids, by decreasing food intake due to central effects, and by directly increasing peripheral insulin sensitivity (128–131). Metreleptin therapy has been shown to increase insulin sensitivity and insulin secretion, to reduce hypertriglyceridemia, hyperglycemia, HbA1c and fatty liver disease, and to improve quality of life (3, 6, 7, 130, 132–134) (**Figure 5**). Other recent studies have shown that metreleptin could also improve cardiac hypertrophy by reducing lipotoxicity and glucose toxicity (135), and decrease mortality risk in patients with lipodystrophy (134). In accordance with the different severity of leptin deficiency, metreleptin therapy seems more efficient in generalized than in partial forms of lipodystrophy (136). In a recent *post-hoc* statistical analysis of two clinical trials conducted at NIH, HbA1c improved by a mean 2.16 percentage point after 12 months of treatment with metreleptin in patients with generalized forms of lipodystrophy (n=59), but only by a mean 0.61 percentage point in patients with partial lipodystrophy (n=36) (137). Therefore, in patients with partial lipodystrophies, metreleptin is recommended in selected patients, with severe hypoleptinemia, HbA1c > 8% and/or serum triglyceride > 500 mg/dL, for whom standard treatments have failed to achieve adequate metabolic control (7). Further studies are needed to determine specific predictive factors for metreleptin response in patients with partial lipodystrophies.

Metreleptin therapy is well tolerated in the majority of patients. The dose of metreleptin is adapted to metabolic responses and to tolerance, with particular attention to the extent of weight loss, which is an expected effect of the treatment but should be controlled. Common adverse effects mainly comprise localized skin reaction, pain at injection sites, and hypoglycemia when the decrease of other antidiabetic treatments is not sufficiently anticipated. Very rare cases of lymphoma have been reported in patients with autoimmune forms of lipodystrophy treated with metreleptin, without any established causal relationship with the treatment (138). Although they display only exceptionally neutralizing effects, circulating anti-leptin autoantibodies frequently develop under treatment. Anti-leptin autoantibodies interfere with enzyme-linked immunosorbent assays for serum leptin, complicating the correct interpretation of leptinemia in treated patients. Further studies are needed to identify other consequences of anti-leptin autoantibodies (6).

## Conclusion

Lipodystrophy syndromes are rare and heterogeneous diseases. Their diagnosis is difficult and can be significantly delayed, since adipose tissue is not systematically investigated during clinical exam, and several symptoms are nonspecific (7). Most clinical forms of lipodystrophy remain genetically unexplained. Next generation sequencing technologies with exome or genome analysis will probably allow for discovering new causative genetic variants in the near future, and lead to a better understanding of the pathophysiology in these rare diseases. However, the interpretation of genetic variants is increasingly challenging (44). Closely associated genetic, clinical and fundamental research, as well as broad collaborations are needed to explore new pathophysiological determinants of lipodystrophy syndromes, and improve the care of affected patients.

## Author Contributions

JZ and CVi wrote the original draft. CVa, EC, MA, CS-L, EB, HM, BD, SJ, BF, and IJ reviewed and edited the manuscript and the figures. All authors contributed to the article and approved the submitted version.

## Funding

Our group is supported by the French Ministry of Solidarity and Health, Assistance-Publique Hôpitaux de Paris, Sorbonne University, the Institut National de la Santé et de la Recherche Médicale (Inserm), and the Fondation pour la Recherche Médicale (EQU201903006878), France.

## Conflict of Interest

The authors declare that the research was conducted in the absence of any commercial or financial relationships that could be construed as a potential conflict of interest.

## Publisher’s Note

All claims expressed in this article are solely those of the authors and do not necessarily represent those of their affiliated organizations, or those of the publisher, the editors and the reviewers. Any product that may be evaluated in this article, or claim that may be made by its manufacturer, is not guaranteed or endorsed by the publisher.

## Glossary

**Table:**

Acromegaloid features	clinical signs typically associated with acromegaly, due to growth hormone overproduction, which may also be observed in insulin resistance syndromes, i.e. broadened extremities and coarsening of facial lines with widened and thickened nose, prominent cheekbones, and enlarged forehead
Android fat distribution/morphotype	body fat distribution characterized by a predominant localization of adipose tissue in abdominal and upper thoracic regions
Autoinflammatory diseases	heterogeneous group of diseases characterized by recurrent inflammatory episodes with fever and increased inflammatory markers, due to a dysregulation of innate and/or adaptive immunity
Bichat fat pads	subcutaneous facial fat of the cheeks and temples
Cellular lipotoxicity	cellular dysfunction mediated by the accumulation of fatty acids derivatives
Cushingoid features	clinical signs typically associated with Cushing syndrome, due to corticosteroid overproduction, which may also be observed in some partial lipodystrophy syndromes, i.e. rounded face, doubled chin, supraclavicular, axillar and dorsocervical fat accumulation (buffalo hump)
Diabetic cardiomyopathy	myocardial dysfunction in the absence of overt clinical coronary artery disease or valvular disease observed in patients with diabetes mellitus
Liver steatosis	lipid accumulation in the liver which may lead to liver dysfunction, inflammation and fibrosis
Metabolic inflexibility	inability to adapt substrate oxidation to nutrient availability and hormone regulation – for example, in insulin resistance states, inability to switch from lipid to carbohydrate oxidation upon insulin stimulation
Multisystem diseases	disorders that affect several organs or tissues involved in specialized functions or in different physiological systems (i.e. cardiovascular system, endocrine system, central or peripheral nervous system, digestive system, immune system …)
Osteolysis	destruction of bone tissue
Osteosclerosis	localized or diffuse increased density of bone tissue
Progeroid syndromes	heterogeneous group of rare diseases characterized by clinical features of accelerated aging
Pseudo-lipomatous regions/pseudo-lipomas	unencapsulated masses of adipose tissue which can be clinically misdiagnosed as lipomas (encapsulated benign tumors of fatty tissue)
Pseudo-osteopoikilosis	numerous islands of osteosclerosis in the skeleton detected as spotted lesions on x-ray pictures
Segmental lipoatrophy	loss of adipose tissue involving a part of the body
Trophic skin disorders (observed in progeroid syndromes)	skin atrophy, dry and/or rigid skin with increased visibility of veins, changes in color and temperature, and/or impaired wound healing
